# Stroboscopic Surface Thermal Lensing for Fast Detection of Thermal Defects in Large-Scaled Coating Films

**DOI:** 10.1155/2015/942138

**Published:** 2015-05-28

**Authors:** Chunxian Tao, Dawei Zhang, Ruijin Hong, Zhongfei Wang

**Affiliations:** Shanghai Key Laboratory of Modern Optical System, Optical Instruments and Systems Engineering Research Center, Ministry of Education, University of Shanghai for Science and Technology, Shanghai 200093, China

## Abstract

A stroboscopic surface thermal lensing (SSTL) system for the fast detection of thermal-induced defects in large-scaled optical coating films was constructed. The SSTL signal was generated by a set of double-modulators and captured by a high speed matrix camera, respectively. The spot size of both pump laser and probe laser expanded for larger detection area was finished in a single step. Based on the STL technique, both the mapping of amplitude and the phase of SSTL signal on the whole area of the coatings can be achieved simultaneously.

## 1. Introduction

In the laser-induced damage of coating films, absorption of the radiated laser energy is the primary cause of damage [1, 2]. Thermal characterization of the radiated coating films is meaningful in defect identification and a supplementary means for laser-induced damage threshold enhancement [3]. Mathematical analysis and numerical simulations of heating processes of photothermal effect have been intensively studied [4–7], both for the interpretation of measurements signal and for the development of measurement methods, applied in the design and quality control [8–10]. Until now, the photothermal microscopy has been used for absorption and defects detection in crystals or optical coatings. The surface thermal lensing technique has become a popular method with the merit of high sensitivity. However, the STL has been used only in small sample due to its limited speed and stability. The requirements of high speed and efficiency in measurement of large-scaled optical coating films are becoming more and more urgent.

The lock-in theory is widely used in thermography for nondestructive detection, such as lock-in thermal IR imaging or thermal wave lock-in imaging [11–13]. With the help of IR or visible charge coupled device (CCD) camera, the optical lock-in has been brought out and used in topography imagery [14, 15]. Due to the advantage of whole field detection, the optical lock-in has the potential of fast and effective detection. In this paper, based on the optical lock-in method, a stroboscopic surface thermal lensing (STL) system was constructed and introduced into the fast defects identification in large-scaled coating films. By both pumping and probing beam modulation configuration, the mapping of the STL signal can be captured by an array CCD camera. Both the amplitude and the phase of the modulated thermal response given information on the thermal-physical properties of the sample were investigated.

## 2. Principle of Stroboscopic Surface Thermal Lensing

When the thin film coating is irradiated by a pump light, the absorption of optical energy by the film sample causes local heating, which produces special physical phenomena in the sample. The surface thermal lensing effect induced by heat absorption in coating film can be interpreted as a spatial distribution of the refraction index dependent on the energy distribution of the irradiation light beam. The fundamental mode laser of Gaussian beam or flat-topped light beam is used as the pump light [16, 17]. Based on the Fresnel diffraction theory, the reflected electric field of probe beam (fundamental Gaussian mode) on the detection plane is expressed as [9]

**Figure 1:**
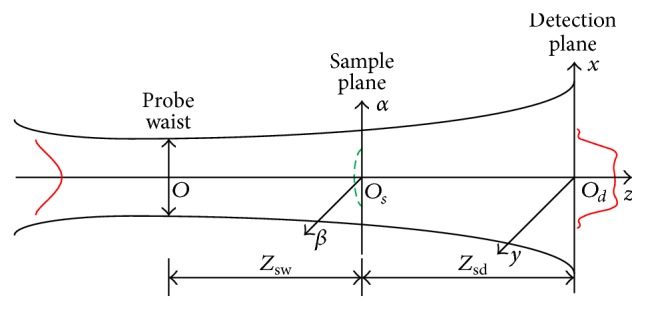
Schematic diagram of stroboscopic surface thermal lensing.

(1) Er,t=ceiarctan⁡zsw/f+ik−zsw+zsd⁡iλzsdω1·∫−∞∞∫−∞∞e−ik/2qα2+β2−2ikur,t+ik/2zsd⁡x−α2+y−β2dα dβ,

where *c* is a constant, *k* is the wave number, *λ* is the wavelength, *z*_sw_ is the distances from the probe beam waist to the sample plane, and *z*_sd_ is the distances from the sample plane to the detection plane. The heat deformation due to the absorption of the film is expressed as *u*(*r*, *t*) and the central highness is proportional to the desired absorptance *A*. The schematic diagram of the surface thermal lensing is shown in Figure 1.

**Figure 2:**
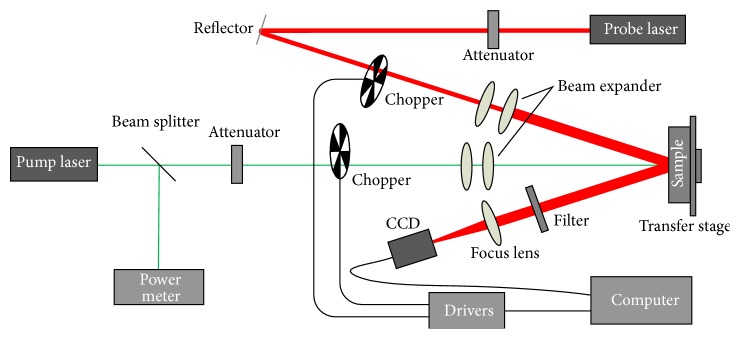
Schematic diagram of stroboscopic surface thermal lensing and signal process.

Although there are many methods to demodulate the STL signal as described in (3), it would be desirable to have a synchronous detection to all the points in a larger excited area. To this purpose, optical lock-in system of stroboscopic photography with large pump laser spot size was constructed with the matrix camera. There is an additional modulator 2 of probe light used for realization of the double-modulation of optical-locking in. And thus the stroboscopic signal of the STL can be captured by the CCD, which is controlled by the same counter in the sequence. The schematic of stroboscopic surface thermal lensing is shown in Figure 2.

According to the correlation function of optical lock-in signal, the lock-in device multiplies the observed signal with two reference functions at the frequency *f* 10 Hz, respectively, which are quadrature with each other. And then it integrates both products by integrator to give the in-phase (*v*_*s*_sin⁡*θ*_*s*_) and quadrature phase (*v*_*s*_cos⁡*θ*_*s*_) components [18]. Therefore, *θ*_*s*_ and *v*_*s*_ of the detecting signal are derived, which represents the RMS amplitude and the phase difference, respectively. In our SSTL system, the modulation frequency *f* and phases difference Δ*φ*(*t*) between the probe and pump are controlled by the counter driver (NI, PCI6601). The digital phase-shift device composed of two electronic fast shutters is triggered by specified pulse trains generated by a digital pulse generator.

The four-point correlation meets our requirement, in virtue of popular modulation mode, more noise-immunity, online operation and high efficiency. Generally, in sinusoidal wave four frames of equidistant signal data are enough to derive the phase shift *θ*_*s*_ and the amplitude *v*_*s*_ as

**Figure 3:**
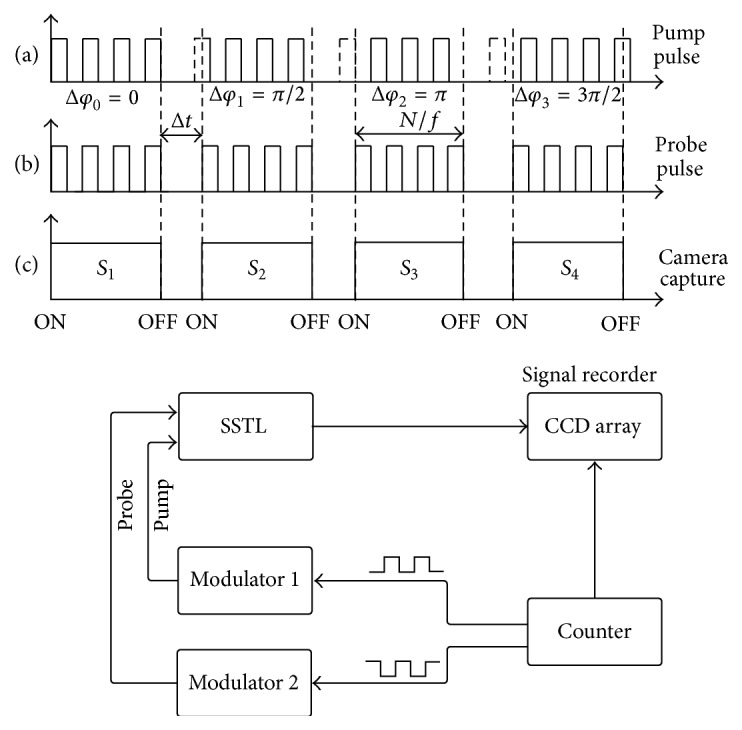
Pulse sequence of double-modulation of four-point correlation for stroboscopic surface thermal lensing.

(2) θsarctan⁡S1−S3S2−S4,vs=S1−S32+S2−S42,

where *S*_1_, *S*_2_, *S*_3_, and *S*_4_ are stroboscopic STL signals on the pixel (*x*, *y*) of the film with phase shift *π*/2, as shown in Figure 3(c). To obtain these four kinds of signal, the phase shift between the pump and probe triggers pulse sequences specified as in Figures 3(a) and 3(b). And thus at least four phase differences, called in-phase and quadrature phase, are obtained:

(3) $$\begin{array}{c} \Delta \phi \left(\;t\;\right)=\phi_{\text{probe}}-\phi_{\text{pumb}}=2\pi ft_{i}=\left\{\begin{array}{cc} 0, & \pi \\ \frac{\pi }{2}, & \frac{3\pi }{2}. \end{array}\right. \end{array}$$

To obtain maximum output, phase differences of integral multiple quarter wave are the best choice. Consequently, the selected four detecting time points of CCD camera are

(4) $$\begin{array}{c} t_{i}=\left\{\begin{array}{cc} 0, & \frac{1}{2}f\\ \frac{1}{4}f, & \frac{3}{4}f. \end{array}\right. \end{array}$$

**Figure 4:**
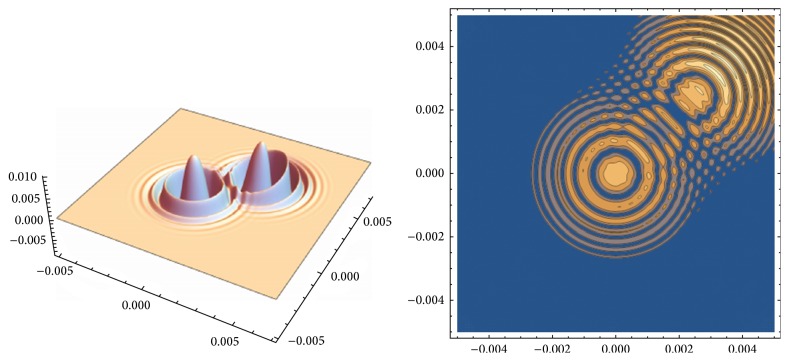
Mapping of the stroboscopic surface thermal lensing in large-scaled radiation area.

The theoretical calculation of the SSTL measurement distribution based on this system is shown in Figure 4. Two typical absorption defects (Au particles in nm scale) are embedded in the subsurface of the Ta_2_O_5_ coating film. The SSTL has the ability of identifying the defects on the whole laser pump field at a time and provides a great help for measurement of large-scaled optical films. The test area is about 10 times as much as that of the single point of STL, determined by the distance between the laser source and the sample, the energy density, and the beam expander. This mapping process is a development of (3) to the whole radiation area of pump beam, such as top-hat beam [19]. Compared with the single pixel detection, the image sensor of camera is made up of amounts of small photosensor elements; each of them may act as the required lock-in detector array. Consequently, large amounts of points of the sample can be detected simultaneously.

## 3. Analysis of Detection Errors

This solution of (3) can be interpreted as strobe frequency or stroboscopic photography. When an oscillatory sample at frequency *f* with initial phase *α* is irradiated by a pulsed light source at *f*_pulse_, once the condition Δ*f* ≡ *f*_pulse_ − *f* = 0 is satisfied, the phase *α* of the vibration will be frozen. And, thus, the image of the sample with this fixed phase can be repeatedly captured by the CCD camera. Consequently, first, the demand on high capture speed can be overlooked. Secondly, integration in multiple capture cycle is helpful to reduce random noise.

The measuring accuracy is another key character for measurement. The captured images of the sample in *N* cycles can be expressed as the product of the vibration function of the pumping probe beam and the pulse array of the probing beam:

(5) s′∫0N/f1+ei2πft+α2∑0Nδt−t0dt=1+ei2πft0+α2,

where *t*_0_ is the point in the capturing time. Since the integration is in a period of time pulse width Δ*t*, the practical signal can be expressed as

(6) s∫t0t0+Δts′dt0=12∑0NΔt+ej2πfΔt−12jπfej2πft0+α≈∑0N1+ej2πft0+α2Δt.

From (6), we find that the measuring accuracy is only a function of the pulse width. With the fixed modulation frequency, the pulse width of image acquisition time should be reduced as much as possible, which can greatly improve the accuracy of measurement.

## 4. Conclusions

The stroboscopic and the surface thermal lensing was integrated for fast defects detection of optical coatings and crystals. Using a matrix CCD camera with high frame rate, the mapping of amplitude and phase of SSTL on a large area of thin coatings could be obtained simultaneously. The stroboscopic STL technique, being proposed to realize fast measurement of weak absorption and defects identification in large-scaled optical coating films, can provide a new and effective diagnostic tool for laser conditioning on large-scaled thin coating films.
